# ADNEX Model-Based Diagnosis of Ovarian Cancer Using MRI Images

**DOI:** 10.1155/2021/2146578

**Published:** 2021-08-18

**Authors:** Bin Liu, Jianmei Liao, Wenli Gu, Junyan Wang, Guozhang Li, Liang Wang

**Affiliations:** ^1^Department of Radiology, Kunming Yan'an Hospital, Kunming 650051, China; ^2^Chengdu Women's and Children's Central Hospital, School of Medicine, University of Electronic Science and Technology of China, Chengdu 610031, China; ^3^Department of Gynecology, Ningxia Medical University General Hospital Cardiovascular and Cerebrovascular Disease Hospital, Yinchuan 750001, China; ^4^Department of Radiology, Wuhan Sixth Hospital, Affiliated Hospital of Jianghan University, Wuhan 430015, China; ^5^Department of Radiology, Taian Children's Hospital, Taian 271000, China

## Abstract

This exploration aims to investigate the important role of magnetic resonance imaging (MRI) in the diagnosis of ovarian cancer under the ADNEX. From March 2017 to December 2019, 84 patients with ovarian cancer confirmed by pathological operation were selected as the research objects. The consistency of ADNEX, MRI, and ADNEX*∗*MRI in the diagnosis and staging of ovarian cancer was calculated separately. SPSS 26.0 statistical software was used to compare the accuracy, sensitivity, specificity, and diagnostic value of the two diagnostic methods. The results show that the accuracy and sensitivity of ADNEX are 78.6% and 93.2%, respectively. The accuracy and sensitivity of MRI are 81.2% and 89.4%, respectively. There is no significant difference between the two methods (*p* < 0.05). The overall consistency rates of ADNEX*∗*MRI, MRI diagnosis, and ADNEX for ovarian cancer staging are 94.2%, 74%, and 65.4%, respectively. There was a significant difference (*p* < 0.05). ADNEX*∗*MRI and MRI diagnosis were compared with each stage of ADNEX. There is a significant difference between the second and fourth stages (*p* < 0.05), and there is also a significant difference in the fourth stage (*p* < 0.017). It is concluded that MRI diagnosis of ovarian cancer based on ADNEX is superior to ADNEX and MRI examination alone, which provides a certain reference value for clinical staging of ovarian cancer.

## 1. Introduction

With the continuous development of medicine, ovarian lesions have attracted more and more attention in the medical field due to their various types, high incidence, and complex tissue composition. The etiology of ovarian cancer is unknown. The known risk factors of ovarian epithelial cancer originating from epithelial cells are smoking, infertility, postmenopausal women, history of ovulation-promoting drugs, and family history of breast cancer or ovarian cancer. Ovarian cancer mostly occurs in postmenopausal women, but there are also many young people [1]. Because of late detection, patients often miss the best opportunity for treatment. How to diagnose and treat ovarian cancer early has always been a difficult problem people need to overcome. Owing to the strong subjectivity of gynecological examination and the inability to judge the surrounding infiltration, many assistant methods for diagnosis of ovarian tumors have emerged as the times require. Major noninvasive imaging examinations include conventional ultrasound, three-dimensional ultrasound, color and energy Doppler ultrasound, computed tomography (CT), magnetic resonance imaging (MRI), and positron emission tomography (PET) [2].

The diagnostic rate of ovarian tumors has been significantly improved with the continuous updating of examination techniques, but there are some limitations in the use of a single technique. According to the data at home and abroad, ADNEX has high diagnostic accuracy and negative predictive value. Clinical studies have shown that subjective ultrasound evaluation of ovarian tumors depends on the experience of the examiner, and experienced ultrasound physicians have the same advantages as diagnostic model indicators, so that ADNEX can obtain satisfactory results [3, 4]. The ADNEX calculates the possible nature and stage of tumors according to the related clinical and ultrasonographic indicators of patients, including age, CAl2 level, maximum diameter of lesions, and ratio of solid components [5].

Second, because of its stable image quality and clear signs of metastasis, MRI has been widely used in the diagnosis of other pelvic diseases. The structure of MRI image is clear, which can clearly reflect the anatomical shape, accurate location, and intuitive signs of metastasis of the lesion through the relationship between surrounding tissues, and provides strong evidence for the selection of clinical stages and surgical methods. The features of ovarian cancer examined by MRI mainly involve three aspects. First, the masses can be early cystic, solid, and cystic in the middle and late stages, with the maximum diameter often exceeding 3 cm and the thickness of septum and cyst wall more than 3 cm. Second, there are often papillary protuberances on the wall, often accompanied by ascites. Third, when the peritoneum or pelvic cavity invades, the corresponding signs of metastasis can be seen, such as peritoneal thickening, pelvic enlarged lymph nodes, and obvious angiogenesis of tumors. Once the signs of metastasis are found, the scope of examination should be expanded [6, 7].

To qualitatively stage ovarian tumors, Assessment of Different NEoplasias in the adneXa (ADNEX) is proposed. In this model, computer software and prospective methods are used to study the adnexal mass in women. Compared with the current ovarian cancer examination methods, the unique advantage of this model is that it can evaluate the tumor staging, and it is inexpensive, which can save medical resources and reduce the economic burden of patients. At present, in China, there is a lack of exploration on the clinical value of the model.

Therefore, in this exploration, ADNEX is innovatively combined with MRI, aiming at discussing the diagnosis of malignant ovarian cancer and its staging based on ADNEX, analyzing its effectiveness in the diagnosis of ovarian cancer, comparing the advantages of the combination of the two characteristics, so as to guide clinical rational and correct selection of examination means, and provides help for disease staging and treatment.

## 2. Materials and Methods

### 2.1. Research Object

The subjects are 200 patients with suspected ovarian cancer admitted to Shandong Province Linyi People's Hospital from March 2017 to December 2019. 116 cases are excluded according to the exclusion criteria. Finally, 84 cases of ovarian cancer are confirmed by surgery and pathology. All patients receive ultrasound and magnetic resonance examination, which are confirmed by pathology after operation.

The criteria of case selection mainly involve three aspects. The first one is the complete history of patients. The second one is the first operation and pathological diagnosis of ovarian cancer. The third one is the complete information of the lesions before and after the operation. The information of the lesions before and after the operation includes detailed records of complete ultrasound examination, pelvic magnetic resonance examination of the size, and location of ovarian masses. Postoperative information included tumor size, adjacency, infiltration degree, pathological classification, and so on [8, 9].

**Figure 1:**
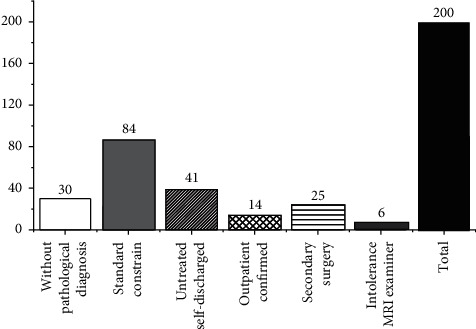
Bar graph of ovarian cancer excluded patients.

The exclusion criteria of cases mainly involve three aspects. Those who have not been confirmed by surgery and pathology are excluded. Those who have been operated twice are excluded. Those who have not been discharged by themselves and those who have been diagnosed outside the hospital are excluded. Those who have been affected by malignant tumors of other origins in the retroperitoneum and pelvic cavity are excluded. Those who are equipped with metal instruments and intolerant of magnetic resonance imaging are excluded [10], as shown in Figure 1.

### 2.2. Inspection Instruments and Methods

First of all, it is necessary to do ultrasound examination for selected patients. Philips color ultrasound diagnostic instrument IU Elite is used. The preparation and scanning conditions consist of three levels. The first one is supine position and filling bladder properly. The second one is abdominal probe C5-1 and frequency 2–5 MHZ, which are examined by abdominal ultrasound. Third, after emptying the bladder, the transvaginal probe C8–4 V frequency is 5–9 MHZ, which is examined by transvaginal ultrasound (only for married people) [11]. The basic information, blood flow, and infiltration of the mass are observed. When metastasis is suspected, scanning of easily metastatic sites can be increased to focus on the analysis of suspicious lesions.

Then, after ultrasound examination, 5 ml venous blood is taken from all subjects on an empty stomach and placed at room temperature for 30 minutes. After centrifugation, superficial serum is obtained and separated into test tubes. Roche Di, Agnostics GmbH, Mannheim, Germany, is used, and all operations are carried out according to the kit and instrument instructions [12].

Then, the patients are examined with the Philips 1.5T MRI diagnostic instrument. There are four levels of preparation and scanning conditions. The first is supine position, filling bladder. The second is scanning range. Pubic symphysis can reach the iliac spine level or higher (diaphragmatic apex when combined with ascites). The third is to find the lesion by plain scan and then to observe the lesion by enhanced scanning. Machenville contrast agent is used to inject 0.1 mmoL per kilogram through the elbow vein. *T*2WI/FSH : TR/TE = 1650/95 ms. *T*1WI/FSH : TR/TE = 550/15 ms. TR/TE = 4450/125 ms. *T*2-weighted plus presaturated sequence, scanning layer thickness 5.0 mm, is of matrix 256*∗*256. The fourth is the observation content, which is ovarian morphological parameters, surrounding infiltration, contrast enhancement [13].

### 2.3. Use of ADNEX and Pathological Examination

**Figure 2:**
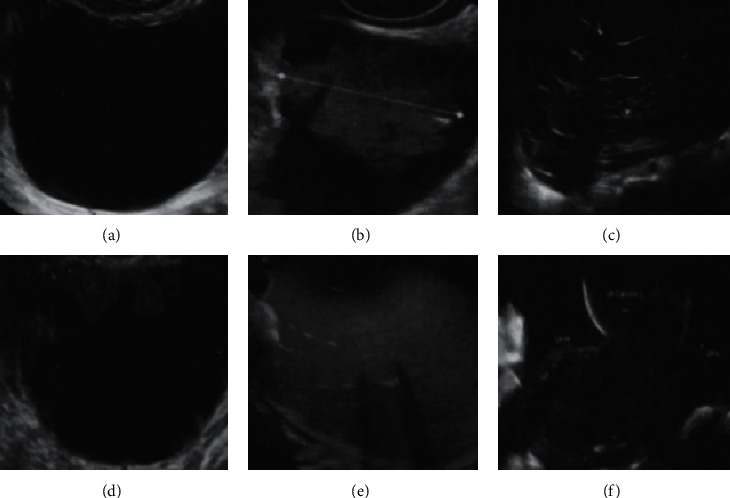
Schematic diagram of the required indicators for ADNEX.

The model includes three clinical indicators and six ultrasound indicators. Three clinical indicators included age, serum CAl25, and types of diagnostic and therapeutic centers (whether they are tertiary medical institutions with cancer diagnostic centers). Six ultrasound indicators are the maximum diameter of lesions, the proportion of solid tissue, whether there are more than 10 cysts, the number of papillary processes, whether there are echoes or not, and whether there are ascites. An example of image acquisition is shown in Figure 2. The access to ADNEX software is http://www.Iotagroup.org/adnexmodel/. Various indices needed by the model are substituted into ADNEX software to automatically generate the possible nature and stage of ovarian tumors [14].

All lesions are confirmed by pathology. The coincidence rates of ADNEX, MRI, and ADNEX*∗*MRI in diagnosis of ovarian cancer are retrospectively analyzed.

Figure 2(a) demonstrates the maximum diameter (mm). Figure 2(b) demonstrates the proportional measurement of solid tissue. Figure 2(c) demonstrates whether the mass exceeds 10 cysts. Figure 2(d) demonstrates the number of papillary processes (0, 1, 2, 3, and >3). Figure 2(e) demonstrates echoes (with or without). Figure 2(f) demonstrates ascites (with or without).

### 2.4. Staging Diagnostic Criteria of Ovarian Malignant Tumors

The staging standards revised by the International Federation of Obstetrics and Gynecology (FIGO) in 2013 are adopted. Phase I: the lesion is confined to one ovary. Stage II: the lesions not only spread in one or both ovaries but also in the pelvis. Phase III: tumor lesions are not only in one or both ovaries but also in peritoneal metastasis outside pelvic cavity and some lymph node metastasis confirmed by cytology or histology. Phase IV: distant metastasis beyond the abdominal cavity [15].

SPSS 26.0 statistical analysis software is used to process all the data. The counting data are tested by the *x*^2^ test. The difference is statistically significant with *p* < 0.05, and the ROC curve is used to test. The two comparisons are based on *p* < 0.017.

## 3. Results and Discussion

### 3.1. Qualitative Analysis of Ovarian Cancer by ADNEX and MRI

**Figure 3:**
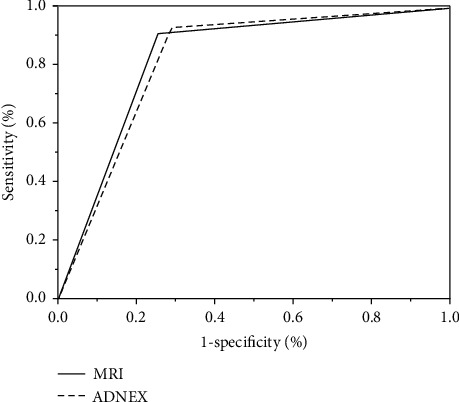
ROC curve.

The collected data are input into ADNEX software for calculation. The accuracy (ACC), sensitivity (SENS), specificity (SPEC), positive predictive value (PPV), and negative predictive value (NPV) of ADNEX are 78.6%, 93.2%, 73.1%, 61.5%, and 95.4%, respectively. The accuracy, sensitivity, specificity, positive predictive value, and negative predictive value of MRI are 81.2%, 89.4%, 75.2%, 62.9%, and 94.4%, respectively. The area under ADNEX curve is 0.821, and the area under MRI examination curve is 0.832, as shown in Figure 3.

MRI is used to examine ACC, SENS, SPEC, PPV, and NPV. There is no significant difference between the diagnostic values detected by MRI and ADNEX, as given in Table 1.

### 3.2. Pathological Results

Cystadenocarcinoma accounts for more than half of the pathological findings of 84 cases of ovarian cancer. Endometrioid adenocarcinoma and Kuckenberg's tumor account for 10.8% of the total, ranking second. The proportion of transitional cancer in 84 patients with ovarian cancer is 9.2%, ranking third. Asexual cell and endodermal sinus tumors account for 6.2% and 2.3%, respectively, as given in Table 2.

### 3.3. Comparison of Three Methods of Pathological Examination

**Figure 4:**
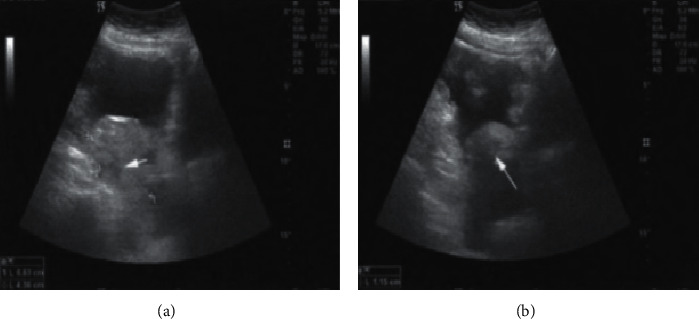
ADNEX for diagnosis of ovarian cancer (conclusion: stage I of ovarian cancer; pathological conclusion: stage I of vegetative cell carcinoma).

**Figure 5:**
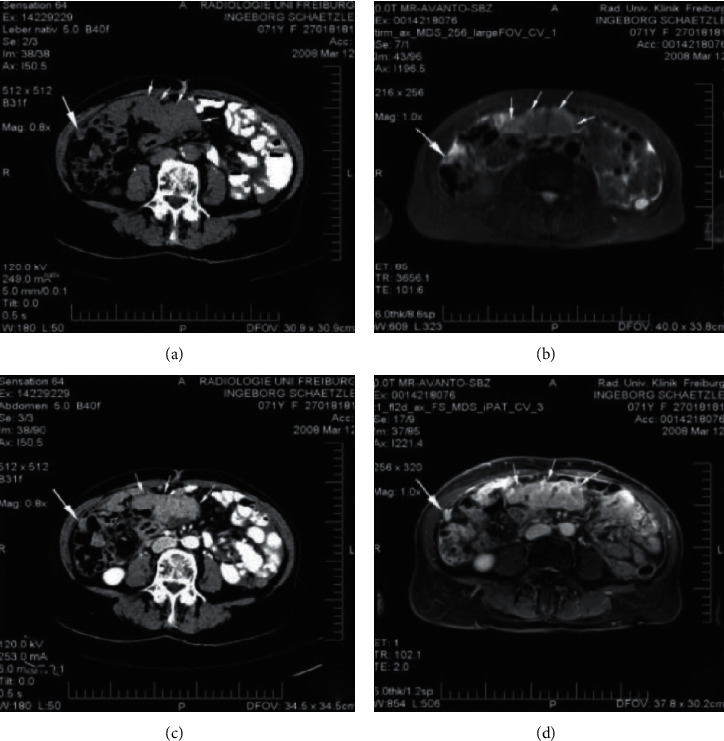
Magnetic resonance diagnosis of ovarian cancer.

**Figure 6:**
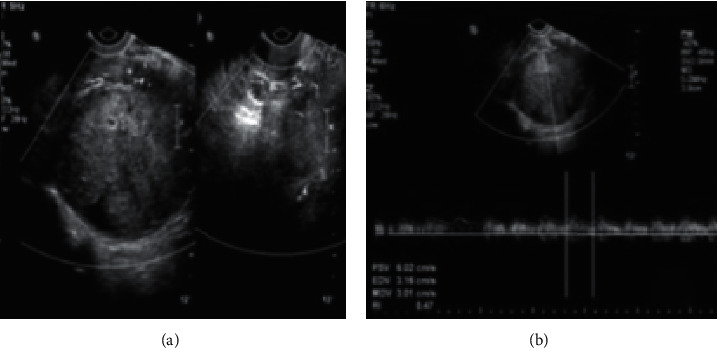
ADNEX combined with MRI in the same case for ultrasound ADNEX examination.

**Figure 7:**
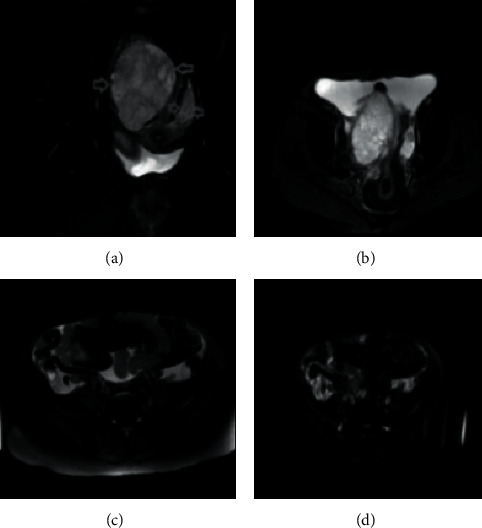
ADNEX combined with MRI in the same case for nuclear magnetic examination.

The coincidence rate and staging coincidence rate of ADNEX, MRI, and ADNEX*∗*MRI in diagnosis of ovarian cancer are compared. As far as the coincidence rate of diagnosis of ovarian cancer is concerned, the total coincidence rate of combined diagnosis is obviously better than that of single diagnosis of ADNEX and MRI, as given in Table 3. As far as the staging of 75 cases of primary ovarian cancer is concerned, the overall coincidence rate of combined diagnosis is obviously better than that of single examination. Specifically, compared with the three methods in each stage, the difference of the three methods in stages II and IV is statistically significant (*p* < 0.05), and the difference of the two comparisons in stage IV is statistically significant (*p* < 0.017), that is, the result of combined examination in stage IV is better than that of single examination, as given in Table 4. ADNEX is used to diagnose ovarian cancer according to its shape, size, and surrounding infiltration. The disease and stage are judged by ADNEX, as shown in Figure 4. The diagnosis of ovarian cancer by MRI is based on peripheral MRI signs and combined with medical history, as shown in Figure 5. The diagnosis of ovarian cancer by ADNEX combined with MRI is based on the symptoms of the two syndromes, as shown in Figures 6 and 7.

The white arrow in Figure 4(a) points to the main mixed echo masses of ovarian solid components. Figure 4(b) shows that ovarian solid components are larger.

Figure 5(a) shows elliptic-like abnormal signal shadow in the lower abdomen-pelvis with a maximum range of 21.68*∗*16.85*∗*12.14 cm, and *T*1-weighted shows low signal (as indicated by the arrow). Figure 5(b) shows *T*2-weighted high signal. Figure 5(c) shows that DWI image shows slightly high signal intensity, uneven signal intensity, thin wall, internal septation, smooth wall, and no obvious wall nodule shadow. The demarcation between the lesion and the right appendix is not clear. Figure 5(d) shows compressive changes in the surrounding intestinal tract and bladder. The sign of intestinal obstruction is indicated by the thick white arrow in the figure. Peritoneal thickening is indicated by a small white arrow.

Figure 6(a) shows a solid hypoechoic mass in the right appendix with a size of 84*∗*102 mm. A solid hypoechoic mass has a size of 44*∗*55 mm in the left side. Figure 6(b) shows CDFI (dot-strip blood flow signals around and inside the mass). RI is 0.47. The depth of the free fluid behind the uterus is about 60 mm.

Figure 7(a) shows mixed signal shadows in bilateral adnexal areas, with cystic and solid masses on the right, solid masses on the left, isohypointense on *T*1, and patchy slightly high signal shadows. Figure 7(b) shows that high signal intensity is dominant in the adnexal masses on both sides, and the lesions on enhanced scan show obvious heterogeneous enhancement. Figure 7(c) shows a small amount of irregular patchy water-like signal shadow in the abdomen and pelvis and mesenteric swelling and exudation. Figure 7(d) shows inguinal lymph node enlargement.

### 3.4. Research and Discussion

The incidence rate of ovarian cancer is only inferior to cervical cancer and endometrial cancer. The pathogenesis is very complex. It often has many complications, such as breast cancer, colon cancer, and gastric cancer. Ovarian cancer has no obvious early symptoms and progresses rapidly. The main clinical manifestations are lower abdominal pain, lower abdominal mass, vaginal bleeding, and abdominal distention. All kinds of early clinical diagnosis are not effective. In this exploration, the combination of ADNEX and MRI in the diagnosis and staging of ovarian cancer is mainly discussed.

In this study, the accuracy of MRI in diagnosis of ovarian cancer is 81.2%. The reason may be due to the different pathological types of ovarian cancer, and the corresponding MRI manifestations of ovarian cancer are also complex and changeable, which is consistent with the research results of Hanchanale et al. [16].

For example, compared with the conventional signs of ovarian cancer, mucinous cystadenocarcinoma in *T*1-weighted sequence also shows high signal intensity. Ovarian endometrioid cancer can occur simultaneously with endometrial cancer, showing mixed signal masses. When combined with hemorrhage, necrosis, and torsion, the mixed signal disorder sonogram shows atypical signs. When the enhanced scan of ovarian masses shows obvious enhancement signs, the enhancement rate alone cannot be used to distinguish benign from malignant lesions. MRI diagnosis of ovarian cancer has its advantages and disadvantages. The total coincidence rate of staging of ovarian cancer diagnosed by MRI is 74.0%. When the pelvic organs are slightly involved, it is difficult to show. The tumors are close to the surrounding structure, and there are few adipose tissues in the space. The tumors less than 1 cm in diameter are difficult to distinguish from the lymph nodes, which may be the causes of staging too high or too low.

The coincidence rate of ADNEX combined with MRI in diagnosis of ovarian cancer (95.4%) is significantly higher than that of ADNEX (80.2%) and MRI (83.1%). For ovarian lesions, transabdominal ultrasound can show the relationship between the lesion and adjacent tissues. Transvaginal ultrasound can avoid gas interference and keep close to the lesion. The internal echo and blood flow can be observed at close range. Ultrasound has the advantages of repeatability, multiangle, and real-time dynamic. Compared with ADNEX, MRI based on fixed slice scan is less flexible than ADNEX, which is consistent with the research results of Joyeux et al. [17], but its spatial resolution and soft tissue resolution are incomparable and irreplaceable. MRI can clearly describe the structure and enhancement pattern of tumors. Ovarian masses have great advantages in differentiating benign and malignant tumors, but there are also shortcomings. Due to the limitations of its own conditions, it cannot be widely used. Therefore, the combination of ADNEX and magnetic resonance imaging in the diagnosis of ovarian cancer can greatly improve the coincidence rate of diagnosis, provide better staging basis, and guide clinical surgical methods and preoperative radiotherapy and chemotherapy.

## 4. Conclusion

The results show that there is no significant difference between MRI and ADNEX in the accuracy and sensitivity of qualitative diagnosis of ovarian cancer. However, the accuracy and sensitivity of ADNEX combined with MRI are higher than that of MRI or ADNEX alone. Generally, because the ovarian position is changeable and it is easy to be affected by intestinal gas, the ultrasound or MRI images are not clear. Therefore, the combination of ADNEX and MRI in the diagnosis and staging of ovarian cancer can not only more accurately observe the size and morphology of ovarian cancer and its relationship with adjacent tissues but also observe the metastasis and invasion of surrounding tissues. Its advantages are as follows. It has the advantages of repeatability, simple operation, real-time dynamic, low cost, and so on, and it also chooses MRI with no radiation and high resolution of soft tissue. The combination of the two greatly improves the diagnostic coincidence rate of ovarian cancer.

Although the principles of ADNEX and MRI are different, the combination of ADNEX and MRI provides a basis for clinical staging. However, there are still some problems in the combination of ADNEX and magnetic resonance imaging, which need to be further diagnosed in combination with clinical practice. It is also the focus of research in the future.

## Figures and Tables

**Table 1:** Comparison of ADNEX and MRI to identify qualitative accuracy of ovarian cancer (%).

Diagnostic value	ADNEX	MRI examination
ACC	78.6	81.2
SENS	93.2	89.4
SPEC	73.1	75.2
PPV	61.5	62.9
NPV	95.4	94.4

**Table 2:** Results of 84 cases of ovarian cancer pathology.

Pathological type	Quantity	Percentage (%)
Cyst adenocarcinoma	50	60.5
Endometrioid adenocarcinoma	8	10.8
Borderline cancer	7	9.2
Kuckenberg tumor	10	10.8
Asexual cell tumor	4	6.2
Endodermal sinus tumor	3	2.5
Total	84	100

**Table 3:** Comparison of results of three examination methods for 84 cases of ovarian cancer.

Inspection method	Number of cases	Compliance rate (%)	*X* ^2^	*P*
MRI	67	83.1^△^	23.16	<0.001
ADNEX	65	80.2		
ADNEX*∗*MRI	78	95.4^☆#^		

*Note*. ^△^MRI compared with ADNEX, *p* < 0.017. ^☆^ADNEX*∗*MRI is better than ADNEX, *p* < 0.017. ^#^ADNEX *∗* MR is compared with MR, *p* < 0.017.

**Table 4:** Comparison of three types of examination methods for 75 cases of primary ovarian cancer, *n* (%).

Pathological staging	Number of cases	ADNEX		MRI		ADNEX*∗*MRI		*X* ^2^	*P*
		Number of cases	Compliance rate	Number of cases	Compliance rate	Number of cases	Compliance rate		
I	12	9	72.1	9	79.1	9	91.2	1.31	0.84
II	14	4	45.4	10	78.3	13	85.1	7.51	0.03
III	21	16	70.1	16	77.4	21	91.0	3.62	0.21
IV	28	13	58.3	20	72.6	27	96.0^☆^	14.4	0.001
Total	75	43	65.4	55	74	72	94.2^☆#^	26.1	<0.001

*Note*. ^☆^ADNEX*∗*MRI is better than ADNEX, *p* < 0.017.

## Data Availability

No data were used to support this study.
